# A patient-centered ‘test-drive’ strategy for ankle-foot orthosis prescription: Protocol for a randomized participant-blinded trial

**DOI:** 10.1371/journal.pone.0302389

**Published:** 2024-05-02

**Authors:** Benjamin R. Shuman, Brad D. Hendershot, David C. Morgenroth, Elizabeth Russell Esposito

**Affiliations:** 1 VA RR&D Center for Limb Loss and Mobility, VA Puget Sound Health Care System, Seattle, Washington, United States of America; 2 Department of Mechanical Engineering, University of Washington, Seattle, Washington, United States of America; 3 Seattle Institute for Biomedical and Clinical Research, Seattle, Washington, United States of America; 4 Extremity Trauma and Amputation Center of Excellence, Defense Health Agency, Falls Church, Virginia, United States of America; 5 Department of Rehabilitation, Walter Reed National Military Medical Center, Bethesda, Maryland, United States of America; 6 Department of Physical Medicine & Rehabilitation, Uniformed Services University of the Health Sciences, Bethesda, Maryland, United States of America; 7 Department of Rehabilitation Medicine, University of Washington, Seattle, Washington, United States of America; IRCCS Medea: Istituto di Ricovero e Cura a Carattere Scientifico Eugenio Medea, ITALY

## Abstract

**Background:**

Ankle-foot orthoses (AFOs) are commonly used to overcome mobility limitations related to lower limb musculoskeletal injury. Despite a multitude of AFOs to choose from, there is scant evidence to guide AFO prescription and limited opportunities for AFO users to provide experiential input during the process. To address these limitations in the current prescription process, this study evaluates a novel, user-centered and personalized ‘test-drive’ strategy using a robotic exoskeleton (‘AFO emulator’) to emulate commercial AFO mechanical properties (i.e., stiffness). The study will determine if brief, in-lab trials (with emulated or actual AFOs) can predict longer term preference, satisfaction, and mobility outcomes after community trials (with the actual AFOs). Secondarily, it will compare the in-lab experience of walking between actual vs. emulated AFOs.

**Methods and analysis:**

In this participant-blinded, randomized crossover study we will recruit up to fifty-eight individuals with lower limb musculoskeletal injuries who currently use an AFO. Participants will walk on a treadmill with three actual AFOs and corresponding emulated AFOs for the "in-lab” assessments. For the community trial assessment, participants will wear each of the actual AFOs for a two-week period during activities of daily living. Performance-based and user-reported measures of preference and mobility will be compared between short- and long-term trials (i.e., in-lab vs. two-week community trials), and between in-lab trials (emulated vs. actual AFOs).

**Trial registration:**

The study was prospectively registered at www.clininicaltrials.gov (Clinical Trials Study ID: NCT06113159). Date: November 1^st^ 2023. https://classic.clinicaltrials.gov/ct2/show/NCT06113159.

## Introduction

Ankle-foot orthoses (AFOs) are commonly worn by individuals with lower limb neuromusculoskeletal deficits to overcome mobility limitations, improve stability, and reduce pain during functional movements. For example, AFOs improve walking speed and functional performance outcomes among individuals with traumatic injuries, [1,2], and can facilitate return to duty for some injured Service members and community participation in Veterans [3,4].

Optimal AFO prescription should match the needs and abilities of the user with the functional characteristics of the device. There is scant evidence to guide AFO prescription [5–7], and there are limited opportunities for AFO users to provide experiential input during the process. Currently, the AFO prescription process relies on prescribing clinician intuition, training, experience [8], and qualitative guides provided by manufacturers [9]. While broad categories of AFOs are recommended to restrict movement in specific planes or augment weakness in specific muscle groups, the clinician must ultimately select from a wide variety of options [5,6]. Moreover, there is a lack of objective information on AFO mechanical properties (e.g., stiffness) with inconsistent and qualitative paradigms for stiffness characterization across manufacturers (e.g., sequential numbering, arbitrary units of measurement, different colors). These differences limit a clinician’s ability to compare devices across vendors to match design features of AFOs to a given patient’s needs. Previous studies have often sought to identify the better AFO between two options for specific groups of AFO users [2,10–17]; However, traditional comparative effectiveness studies—by design—are not sensitive to the unique aspects of each user and the fact that different AFO users may have different “best” AFOs.

A vital aspect of a successful AFO prescription is user satisfaction with the device and perceived mobility [18,19]. Thus, there are likely substantial benefits to incorporating user feedback into the AFO prescription process. Moreover, AFO users routinely want and expect their input to be considered in their plan of care [20,21], and doing so can improve outcomes [22]. However, in current clinical practice (for both custom-made and off-the-shelf AFOs) there are limited opportunities for AFO users to try different devices and offer feedback during the prescription process. Lack of familiarity with available options, and failure to include user feedback in the device contributed to dissatisfaction among one-fourth of AFO users [23].

Trials with different devices would afford users the opportunity to experience a range of AFOs and voice their preferences. However, trialing multiple devices, particularly custom devices, can be expensive, time-consuming, and highly labor-intensive. A robotic exoskeleton emulator (AFO emulator), capable of mimicking certain mechanical characteristics of AFOs, could facilitate a more efficient approach to AFO prescription that would enable incorporating user feedback. Recent work using a prosthetic foot emulator allowed participants to ‘test-drive’ prosthetic feet [24,25]. The current AFO study incorporates a similar ‘test-drive’ strategy for AFO prescription, using a customizable AFO emulator (Caplex system, Humotech, Pittsburgh, PA, USA) that is worn around the foot and lower leg. This novel strategy enables the AFO user to provide real-time experiential feedback and may streamline the prescription process and improve downstream outcomes. While the AFO emulator is not meant to replace clinical expertise, it may serve as a tool to augment the prescription of AFOs.

The purpose of this study is to assess the validity of a patient-centered and personalized ‘test-drive’ strategy for AFO prescription in AFO users with lower limb musculoskeletal injuries. The primary aims of this study are to 1) assess whether AFO preference during brief trials of candidate AFOs (either using the emulator or the corresponding actual devices) is predictive of AFO preference, and user-reported and performance-based mobility and stability measures with the corresponding actual AFOs after two-week community trials, and 2) compare function, mobility, and preference outcomes of brief within-laboratory trials between the emulated and actual AFOs.

## Methods and analysis

### Study design

**Fig 1:**
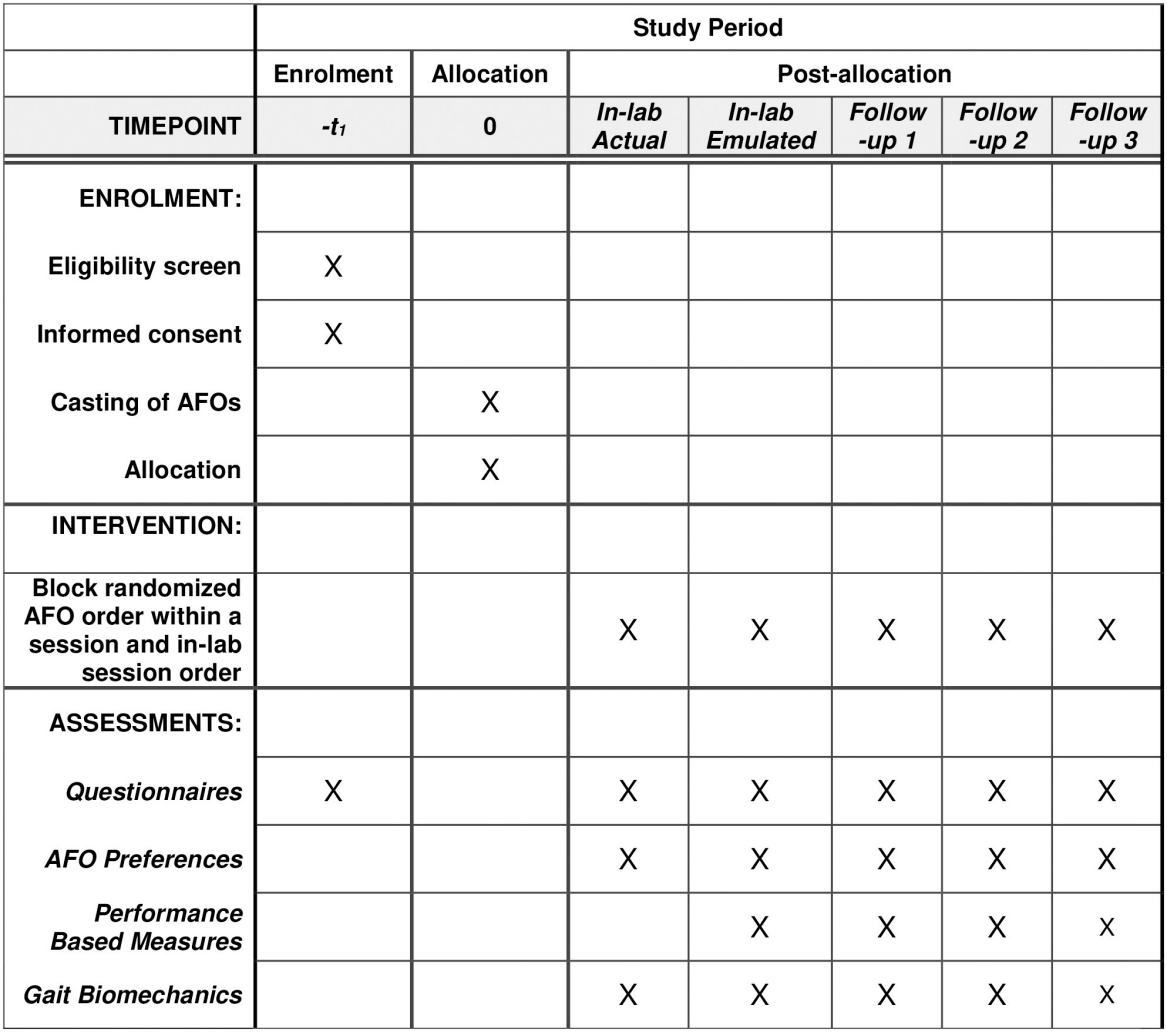
SPIRIT schedule of Enrolment, interventions and assessments for trials with three different ankle-foot orthoses (AFO).

**Fig 2:**
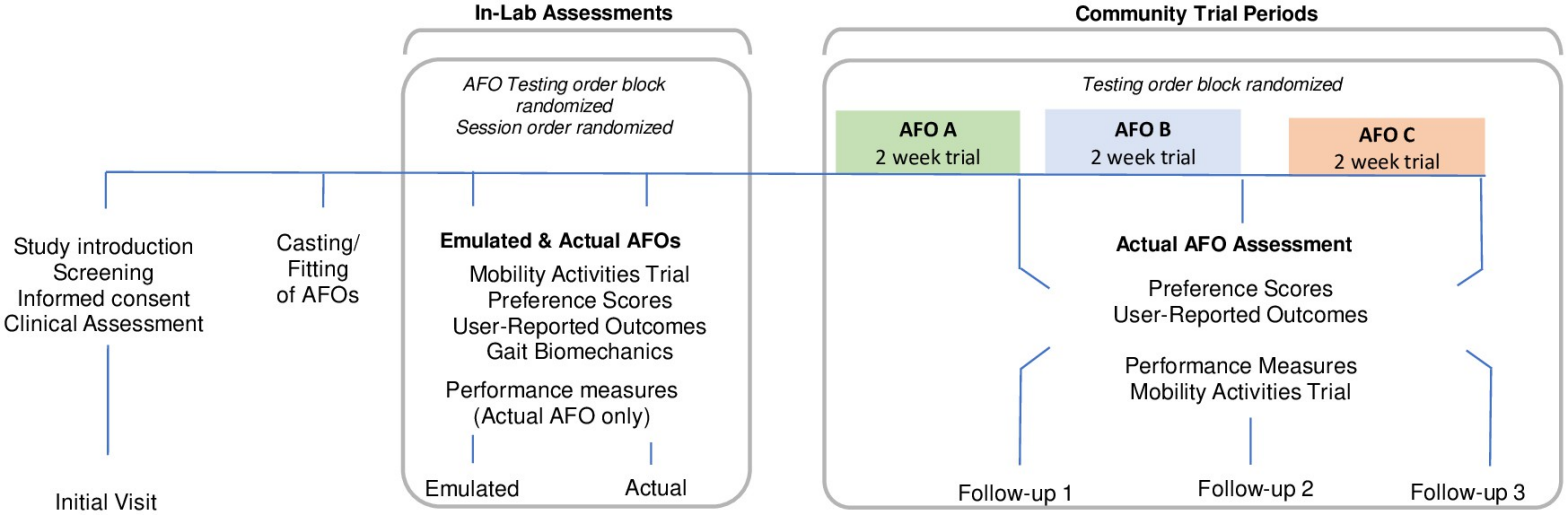
Study overview detailing sequence and timeline for both short-term in-lab evaluations and longer-term community trials with three different ankle-foot orthoses (AFO).

This is a multisite clinical trial with a participant-blinded, randomized, cross-over study design with repeated-measures assessments. Outcomes (user preference, satisfaction, perceived mobility, and performance) will be collected from short-term, within-laboratory trials of emulated and corresponding actual AFOs (Figs 1 and 2). Outcomes from both short-term, in-lab trials (i.e., AFO emulator and actual AFOs) will be compared to longer-term community trials using the actual AFOs. Secondarily, outcomes will also be compared between both emulated and actual short-term in-lab trials.

### Recruitment

We aim to recruit 56 participants (see Statistical analysis for sample size details) with lower limb musculoskeletal injury(ies) who currently use an AFO. Study data collections will occur at two sites: Walter Reed National Military Medical Center and Veterans Affairs Puget Sound Health Care System. AFO users may be identified using the electronic medical records or participant registries and contacted to discuss their interest in participation. AFO users referred by local clinics or who respond to flyers will be provided information on the study and an opportunity to participate, if eligible.

### Inclusion/Exclusion criteria

#### Inclusion criteria

1. Lower extremity injury resulting in the current use of an AFO with at least two-weeks experience.
2. Unlimited community ambulation [26].
3. Age 18–65 years.
4. Body mass between 45–113 kg (based upon emulator constraints).
5. Score less than or equal to 1/5 for average resting pain and less than 2/5 for pain with activity as on the foot wearing an AFO.
6. Foot size between U.S. Men’s 7–13 (based upon emulator constraints).
7. English speaking.
8. Ability to comply with study procedures.

#### Exclusion criteria

1. Known cognitive impairment (e.g., diagnoses such as moderate/severe traumatic brain injury, dementia)
2. Medical conditions that would preclude safe study involvement.
3. Inability to provide written informed consent.
4. Pregnancy (as per self-report).
5. History of neurological impairment that currently limits mobility.
6. Use an assistive device such as a walker or cane.
7. Other conditions that preclude the use of an AFO (e.g., limited ankle ROM)

### Emulator system overview

The AFO emulator system includes an off-board control system and actuator unit, and an ankle-foot exoskeletal end effector (worn by the user) connected with a flexible tether. Benchtop experiments have demonstrated the AFO emulator’s high torque control performance [27–29]. Of note, this system has successfully been used to emulate a broad range of passive, spring-like properties [29], including prosthetic feet [24,25], making it well suited to be programmed to emulate certain mechanical properties of passive-dynamic AFOs without requiring the user to physically change devices. This provides the AFO user with the experience of swapping out different design features in real time via software interface, without the costly and time-intensive trial and error process of comparing actual devices. Shoes can also be quickly swapped with the device to accommodate a range of user foot size. Thus, this setup delivers a powerful exoskeleton end-effector with minimal worn mass and inertia.

The AFO emulator will be programmed to reproduce the sagittal plane torque-angle relationship about the ankle axis of the corresponding actual AFOs. The torque-angle relationship of the AFOs will be measured using the Evaluating Mechanical Properties in Rotating Exoskeletons (EMPIRE) test fixture, the methods for which have been described previously in detail [30]. These data will then be used to program the emulator in an effort to reproduce the experience of wearing the actual AFO.

### Intervention

#### AFO selection

Following consent and enrollment, three different AFOs will be fit to each participant by an orthotist using clinical standards and based on manufacturer recommendations (e.g., foot size, body mass). Additional fitting sessions may be utilized as needed to ensure a good fit to the individual for all three actual AFOs. The three AFOs will vary by participant based upon determination by the study orthotist using clinical judgement, and may include fully off-the-shelf actual AFOs and custom AFOs with configurable componentry. The AFO emulator will be adjusted to fit each participant during the emulated testing session.

#### In-laboratory emulated AFO testing session

**Fig 3:**
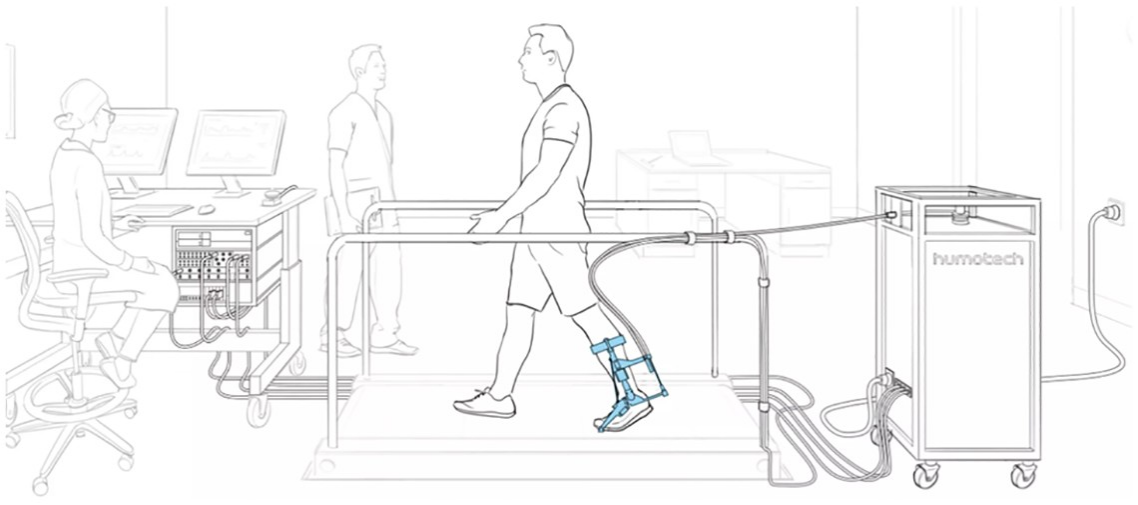
Overview of the emulation “test drive” setup with Ankle-Foot Orthosis (AFO) emulator system (Human Motion Technologies, LLC).

Participants will begin by walking on a treadmill while wearing the AFO emulator (Fig 3) to become accustomed to its use. Participants will wear the AFO emulator’s end effector in place of their clinically prescribed AFO, and the study orthotist will be present to make any necessary adjustments. The AFO emulator parameters will be adjusted by the research team using the control software interface to emulate the sagittal plane torque-angle relationship of the corresponding actual AFOs. Once participants are comfortable in the use of the AFO emulator, participants will trial three emulated AFOs. Participants will complete the Mobility Activities Trial and User-Reported Outcomes (described in the Outcome Measures section below). Between each emulated AFO tested, participants will rest for at least 10 minutes before engaging in the next condition.

#### In-laboratory actual AFO testing session

Participants will walk on the treadmill in each actual AFO condition until comfortable prior to testing. Fitting and adjustments may occur as needed and accommodation time will be given to each participant. Participants will then undergo the same Mobility Activities Trial and User-Reported Outcomes as in the emulated AFO testing session. In the actual AFO testing session, participants will additionally complete a series of performance measures (described in the Outcome Measures section below). Between each actual AFO tested, participants will rest for at least 10 minutes before engaging in the next condition.

#### Two-week community trial sessions

Following the emulated and actual AFO testing sessions, participants will complete three consecutive two-week community trials while wearing each of the three actual AFOs (one for each two-week trial). Participants will be instructed to wear the study assigned AFO instead of their clinically prescribed AFO for all usual activities of daily living. After each two-week trial, participants will return to the laboratory for follow-up evaluation where the User-Reported Outcomes and Performance Tests will be assessed (see details below). The Mobility Activities Trial may also be collected. If needed during the community trials, adjustments (e.g., to padding or alignment), will be made consistent with the standard of clinical care. Any adjustments will be tracked and recorded.

#### Mobility activities trial

The mobility activities include walking on a treadmill at three speeds (based on leg length [27]), up/down a slope, and optionally (if able) running on the treadmill or walking on a stairmill. These activities were selected to represent a range of those that commonly occur in the community environment. Each speed and activity condition will take place for at least 30-seconds and rest will be provided between activities as needed.

### Outcome measures

#### User-reported outcomes

Participants will complete a series of questionnaires throughout the study (Table 1). At intake, participants will be asked about their injury history and self-identified demographics.

**Table 1:** Questionnaire administration schedule.

Questionnaire	Session					
	Initial Visit	In-Lab Actual	In-Lab Emulated	Follow-Up 1	Follow-Up 2	Follow-Up 3
Injury History	x1					
Demographics	x1					
PROMIS Pain Intensity	x1			x1	x1	x1
Pain 5pt		x3	x3	x1	x1	x1
ABC-5	x1			x1	x1	x1
Balance Confidence		x3	x3	x1	x1	x1
QUEST	x1			x1	x1	x1
OPUS—LEFS	x1			x1	x1	x1
OPUS–LEFS (selected items)		x3	x3			
OPUS—Satisfaction	x1			x1	x1	x1
OPRO-M	x1			x1	x1	x1
RPE		x3	x3			
Preference		x3	x3	x1	x1	x1
Brace Comparison		x1	x1			x1
Wearing Time				x1	x1	x1
Feedback						x1

Numbers indicate the repetitions of a given questionnaire within each session (i.e., 1 per tested AFO condition).

**Fig 4:**
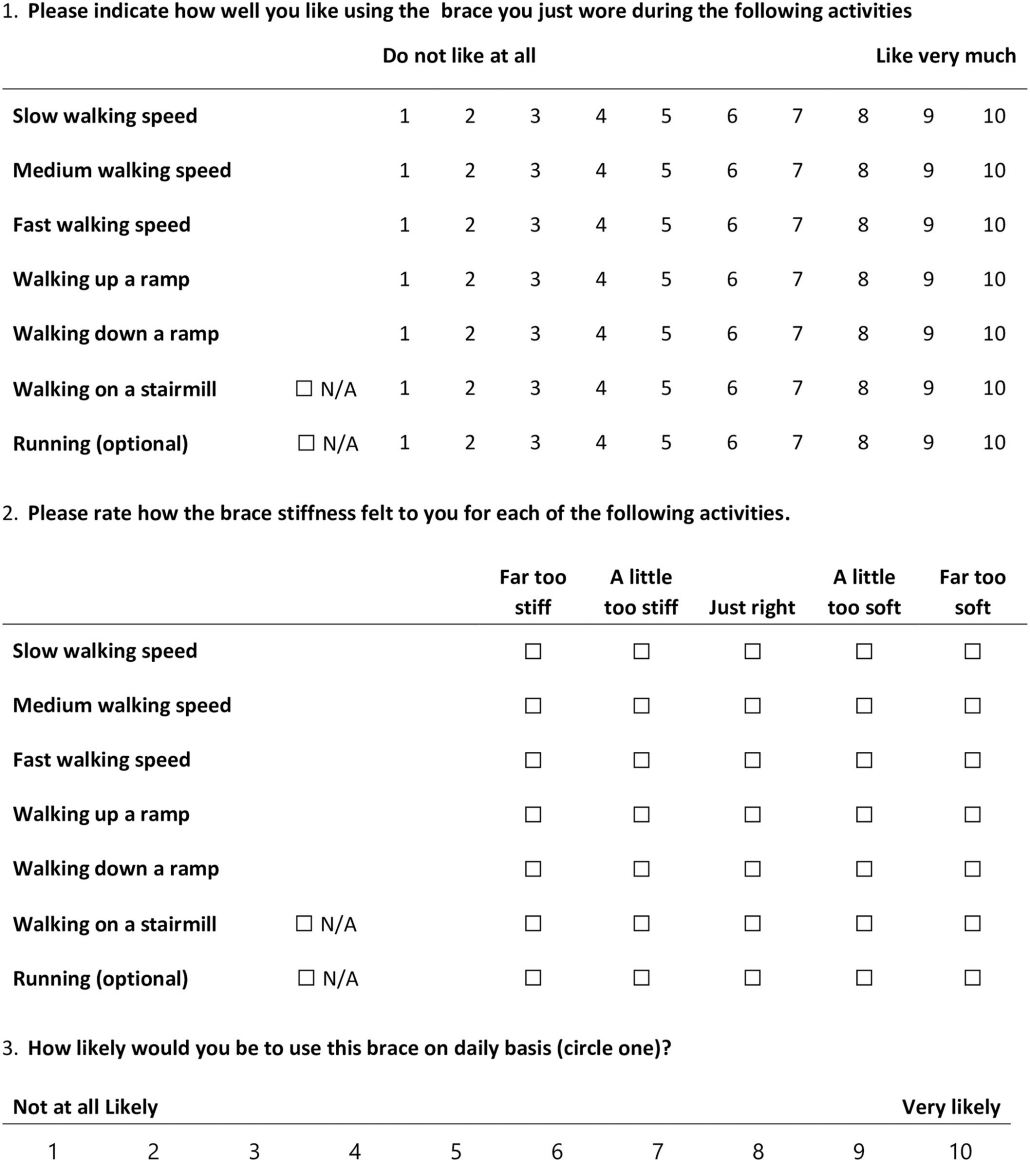
AFO preference questionnaire.

**Fig 5:**
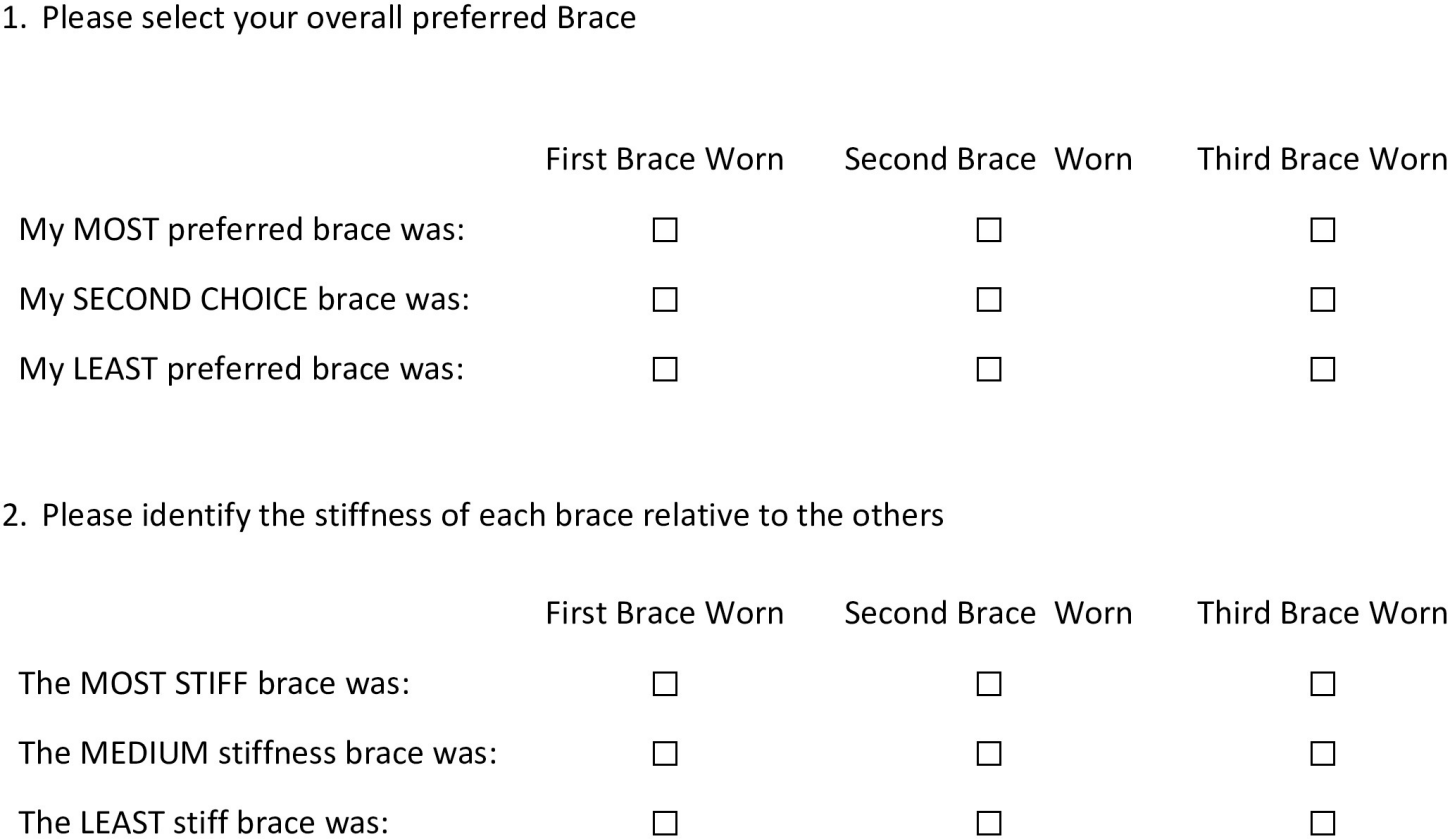
AFO comparison questionnaire.

AFO Preference (Fig 4) is the primary outcome measure that will be assessed using a 10-point scale for each AFO trialed during the emulated in-lab session, the actual AFO in-lab session, and following each community trial. We will also collect preferences for each activity in the Mobility Activities Trial. Additional user assessments of the AFO stiffness ranging from “Far too stiff” to “Far too soft” will be collected. A direct AFO comparison (Fig 5) will be evaluated through ranked preference and perceived AFO stiffness. AFO preference measures are informed by recent work evaluating preference in prosthetic feet [31].

For each item of the Mobility Activity Trial and 2MWT, a rating of Perceived Exertion (RPE) will be recorded on a 6–20 Borg scale [32] and ankle-foot pain (of the AFO limb) will be assessed using an ad-hoc 5-point scale during the in-lab sessions and after each community trial. Pain will also be scored using the Patient-Reported Outcomes Measurement Information System (PROMIS) Pain Intensity subscale [33] at intake and after each community trial, which will be modified to reflect reported assessment of ankle-foot pain on the AFO limb during the previous seven days. The Mobility Activities-Specific Balance Confidence Scale (ABC-5 Scale) will measure participants’ confidence in balance or unsteadiness when performing specific activities of daily living [34]. The full ABC-5 will be used at intake and the end of each community trial. Participants will also be asked to rate their self confidence in their balance for walking on a treadmill, on stairs, and on a ramp (balance confidence questionnaire) during the in-lab sessions and after the community trials. At intake and after each community trial we will also collect the Quebec User Evaluation of Satisfaction with assistive Technology (QUEST 2.0), which will be used to evaluate a user’s satisfaction with assistive technologies [35], and the Orthotic Patient Reported Outcomes–Mobility (OPRO-M) [36] will be used to examine the ability of the participant to complete a range of challenging everyday mobility tasks with their assistive device(s). The Orthotics and Prosthetics Users’ Survey (OPUS) will be used to assess AFO function and satisfaction [37]; in this study, only the following two modules related to the lower extremity will be used: 1) Lower Extremity Functional Status Measure (LEFS) and 2) OPUS- Satisfaction with Devices and Services (questions 1–11 only). Selected items from the LEFS (questions 5, 10, 14, 16, and 17) will be collected during the in-lab emulated and actual AFO sessions while the full LEFS and satisfaction with devices will be collected at intake and after each community trial. Self-reported wear time will be recorded to reflect approximately how many hours the AFO was worn during each day of the community trial period. Additionally, each participant will be asked at the conclusion of their participation in the study to provide any further open-ended feedback they may have about the study or the AFOs used.

#### Performance-based measures

The Two-Minute Walk Test (2MWT) will be used to assess walking ability by asking participants to walk the greatest distance they can in 2 minutes [38]. The Four-Square-Step-Test (FSST) [39] and the Narrowing Beam Walking Test (NBWT) [40,41] will be used to assess dynamic balance. The FSST requires participants to move as quickly as possible across quadrants separated by a 1-inch-high cross-shaped obstacle while always keeping one foot on the floor [39]. The NBWT has participants walk with arms crossed along a 6.71m-length beam of narrowing width until they can no longer maintain balance and step off the beam. The distance of the last foot position on the beam will be recorded [40,41]. The 20-Meter Shuttle Run will also be used to assess power and acceleration/deceleration, [42]. Participants who are not able to run will be asked to walk as quickly as is safely possible to complete the course and may skip the assessment if otherwise not able to complete.

#### Gait biomechanics

During the Mobility Activities Trials, we will evaluate walking biomechanics using retroreflective markers on body joints and segments and ground reaction forces from an instrumented treadmill. As secondary outcomes, we will explore lower limb joint motions, moments, and powers, as well as trunk-pelvis motions, to comprehensively understand human-device interactions and compensations.

### Statistical analysis

To test the ability of the AFO emulator to reproduce the user experience and preferences of wearing actual AFOs, a linear mixed effect (LME) model will be used with all outcome measures considered as continuous response variables. To test whether measurements collected during short, in-lab sessions are good predictors of those same measurements after a longer two-week community trial period, the in-lab data (modeled for both emulated or actual AFOs) modeled as the independent fixed effect, outcome data following the two-week community trial period as the dependent fixed effect, and participant as a random intercept. We hypothesize that AFO outcomes measured during the in-lab sessions (emulated and actual) will be significantly correlated with the experimental AFO outcomes measured during the following the two-week community trial period. Our primary outcome variable is AFO preference with secondary analyses examining user-reported measures.

Similarly, we will test whether the in-lab emulated session outcome measures are good predictors of the same outcomes measured during the in-lab session with actual AFOs. Outcome data from the emulated in-lab session will be modeled as the independent fixed effect, outcome data from the actual in-lab session as the dependent fixed effect, and subject participant as a random intercept. We hypothesize that AFO outcomes measured during the in-lab emulated session will be significantly correlated with the experimental AFO outcomes measured during the in-lab actual session. Our primary outcome variable is AFO preference with secondary analyses examining user-reported measures, functional performance, and gait biomechanics.

Prior to performing the statistical analyses missing data will be evaluated to determine whether systemic origins (e.g. participant age or mobility) or completely at random. Depending on the nature of the missing data we may need to limit the applicability of our findings or attempt to impute the missing data.

#### Power analysis

Our power analysis is based upon pilot data collected for a similar study using prosthetic feet [25] which tested mobility and preference for three participants and two feet between emulated and actual sessions. In that data we found an average increase in 1.1 points in actual preference score per increase in 1 point in emulated score and a 0.3 increase in perceived mobility per increase in 1 point in emulated preference score with residual errors of 3.1 and 1.5 points respectively. Based upon the estimates of slope and residual error from the pilot data, 10,000 datasets were created for a sample size of N = 50. Linear regression (due to the small sample size) was performed on each simulated data set. Power, estimated as the proportion of datasets that rejected the null hypothesis of a slope equal to zero, was 97% for the preference scores and 98% for the mobility scores. To account for a 12% dropout rate experienced in our prior studies on AFO users, a total sample of N = 56 will be recruited.

#### Randomization and blinding

Since the study AFOs will vary between participants based on individual needs, AFOs will be organized within a participant based upon ranked stiffness (slope of the measured torque-angle curve). Participants will be block randomized into three possible AFO testing sequences (e.g., least stiff to most stiff) and each sequence will be evenly divided between participants first receiving the in-lab emulated or in-lab actual session. Study staff will be unblinded to a given testing sequence only after the participant has been consented. To minimize the potential for expectation bias, participants will be blinded to all actual and emulated AFO conditions. AFOs will only be referred to as A, B, C and not by their actual name. Any markings indicating the make and model will be covered or removed. However, it is possible that users may still be able to identify different designs based on color, shape, etc. The study biostatistician will only be presented data coded by AFO condition (A, B, C). If a participant wishes to know the make and model of a certain AFO trialed during the study, it will be disclosed at the conclusion of their participation so they may take that information to their clinical orthotist.

### Ethics approval

This study protocol was approved by the Western Copernicus Group Institutional Review Board (#20224792) and in compliance with all applicable Federal regulations governing the protection of human subjects. Deferral acknowledgements were provided by the local regulatory boards at Walter Reed National Military Medical Center and Veterans Affairs Puget Sound Health Care System.

## Discussion

The goals of this study are to test whether brief trials using an AFO emulator are predictive of the experience of corresponding actual AFOs both in the lab and after two-week community trials. We hypothesize that preference scores and performance-based outcome measures for emulated AFO conditions and in lab corresponding actual AFOs will strongly correlate with preferences measured following the community trials. Further, we hypothesize that that preference scores and performance-based outcome measures for both the in-lab emulated AFO conditions will strongly correlate with those measured in-lab for the corresponding actual AFOs.

Involving AFO users in the clinical decision-making process has been shown to improve functional outcomes [22]. While AFO users have traditionally had limited opportunities to trial devices prior to prescription, a customizable AFO emulator presents a potential opportunity to allow users to experience multiple AFO designs in a time-effective manner. Moreover, an AFO emulator may offer clinicians a new tool to optimize the prescription of AFOs and engineers a method to optimize the theoretical design of future AFOs to better meet the needs of users with lower limb injuries.

There are several limitations to this study. The current AFO emulator can only reproduce measured AFO torques about the ankle axis in the sagittal plane and the location of AFO bending axis has been shown to influence gait performance [43,44]. Although the sagittal plane stiffness is the most prominent AFO feature, frontal and transverse plane stiffness can also impact the AFO user’s performance and preference [6]. The AFO emulator also cannot emulate the footplate stiffness and contour, nor the AFO shape and padding. These serve as confounding factors that can influence both a user’s experience and preference. By also evaluating the ability of in-lab trials with the actual AFOs to predict outcomes after the two-week trial, this study provides the opportunity to examine the extent to which the AFO emulator limitations may impact user preference and provide insight into the clinical utility of short-term trials with actual AFOs. This study is limited to evaluating AFO user with lower limb musculoskeletal injuries and additional studies will be required to generalize the results to additional AFO using populations (e.g. stoke, cerebral palsy, multiple sclerosis).

In conclusion, the selected mobility activities, user-reported outcomes, and performance tests will help elucidate the extent to which in-lab trials using actual AFOs, or a ‘test-drive’ strategy, using an AFO emulator, can be used to predict device function, mobility and preference outcomes following longer-term use for AFO users with lower limb musculoskeletal injuries.

## Supporting information

S1 Fig CONSORT checklist. (DOCX)

S2 Fig SPIRIT checklist. (DOCX)

S1 File (DOCX)
