# Supplementary material for: A patient-centered ‘test-drive’ strategy for ankle-foot orthosis prescription: Protocol for a randomized participant-blinded trial
Source: PLoS One. 2024 May 2;19(5):e0302389. doi: 10.1371/journal.pone.0302389 (PMC11065291; doi:10.1371/journal.pone.0302389)
Supplement: S1 File — (DOCX) [file pone.0302389.s003.docx]

Optimizing Ankle-Foot Orthotic Prescription Using an Emulation Test‐Drive Strategy

Funding Agency: Department of Defense - CDRMP

Version 3; 07/20/2023

Abstract

Provide a summary of the study (recommended length: less than 500 words).

Ankle foot orthoses (AFOs) are often necessary to overcome mobility limitations related to lower limb neuromusculoskeletal injury. While the availability of AFOs for improving function is expanding, options remain limited and especially relative to ankle-foot prostheses. There is a critical need to better optimize AFO designs and provide evidence-based tools to guide their prescription. The proposed study utilizes an exoskeleton capable of emulating AFOs by modulating the magnitude and/or timing of torque.

We aim to (1) test the ability of the AFO emulator to reproduce the user experience of wearing commercially available (‘actual’) AFOs during different in-lab activities (walking, running, stair climbing; level ground, ramps) in individuals with lower limb musculoskeletal injury. This aim will determine if the proposed AFO emulator framework can provide an option for quickly exploring multiple devices in a short period of time. We aim to (2) test the ability of a short-term, in-laboratory evaluation of function, mobility, and preference with the AFO emulator to predict those same outcomes longer-term (i.e., after 2 week use in the community) in AFO users with lower limb musculoskeletal injury. This aim will determine the predictive ability of a brief test-drive strategy using emulated AFOs to identify function, mobility, satisfaction, and preference at followup after two weeks of use in the community environment with corresponding actual AFOs. We aim to (3) allow the user to self-tune AFO emulator stiffness settings while walking and compare function, mobility, preference to the clinically prescribed AFO. This aim will determine if an AFO test drive strategy can be used to better optimize user satisfaction and patient reported outcomes relative to the current standard of care.

We will recruit up to 56 individuals with lower limb musculoskeletal trauma who are currently using an AFO. Participants will first undergo a short-term assessment of three emulated and actual AFOs over two in-lab sessions. Subsequently, participants will complete a community trial with the actual AFOs (2 weeks each). Patient reported outcomes, including user preference, an activities trial, and performance tests (for the actual AFOs only) will be recorded at each assessment. Patient reported outcomes and activities trial will also be recorded for the user self-tuned emulator session.

List of Abbreviations

2MWT - Two-Minute Walk Test

ABC - Activity Balance Confidence Scale

AFO - Ankle Foot Orthosis

AIC - Akaike Information Criterion

CDMRP – Congressionally Directed Medical Research Programs

CITI - Collaborative Institutional Training Initiative

CLiMB - Center for Limb Loss and MoBility

CS - Compound Symmetric

CRADA - Cooperative Research and Development Agreement

DoD - Department of Defense

EACE - Extremity Trauma and Amputation Center of Excellence

ESR - Energy Storing and Returning

FSST - Four square step test

HIPAA - Health Insurance Portability and Accountability Act

HJF - Henry Jackson Foundation

HRPO - Human Research Protection Office

HSD - Honest Significant Difference

ID - Identification

IDEO - Intrepid Dynamic Exoskeletal Orthosis

IRB - Institutional Review Board

LME - Linear Mixed Effect

NCOPE - National Commission on Orthotic and Prosthetic Education

NBWT - Narrowing Walking Beam Test

O&P - Orthotics and Prosthetics

OIF - Operation Iraqi Freedom

OND - Operation New Dawn

OEF - Operation Enduring Freedom

OPUS - Orthotics and Prosthetics Users’ Survey

PHI - Protected Health Information

PI - Principal Investigator

PII - Personally Identifiable Information

PROMIS - Patient-Reported Outcomes Measurement Information System

QUEST - Quebec User Evaluation of Satisfaction with Assistive Technology

RPE - Rating of Perceived Exertion

SAE - Serious Adverse Event

SIBCR – Seattle Institute for Biomedical and Clinical Research

TUG - Timed Up and Go

U-SAE - Unexpected Serious Adverse Event

USAMRMC - U.S. Army Medical Research and Materiel Command

VA - Department of Veterans Affairs

VAPSHCS - VA Puget Sound Health Care System

VHA - Veterans Health Administration

WRNMMC - Walter Reed National Military Medical Center

Contents

[Protocol Title: 7](#_Toc338328603)

[1.0 Study Personnel 7](#_Toc338328604)

[2.0 Introduction 7](#_Toc338328605)

[3.0 Objectives 10](#_Toc338328606)

[4.0 Resources and Personnel 11](#_Toc338328607)

[5.0 Study Procedures 13](#_Toc338328608)

[5.1 Study Design 13](#_Toc338328609)

[5.2 Recruitment Methods 14](#_Toc338328610)

[5.3 Informed Consent Procedures 17](#_Toc338328611)

[5.4 Inclusion/Exclusion Criteria 17](#_Toc338328612)

[5.5 Study Evaluations 18](#_Toc338328613)

[5.6 Data Analysis 24](#_Toc338328614)

[5.7 Withdrawal of Subjects 26](#_Toc338328615)

[6.0 Reporting 26](#_Toc338328616)

[7.0 Privacy and Confidentiality 27](#_Toc338328617)

[8.0 Communication Plan 27](#_Toc338328618)

[9.0 Information Security and Data Storage/Movement 30](#_Toc338328619)

10.0 References…………………………………………………………………………….………… .9

# Protocol Title: Optimizing Ankle-Foot Orthotic Prescription Using an Emulation Test‐Drive Strategy

# Study Personnel

**Overall Project Principal Investigator**: Brad Hendershot, PhD, Walter Reed National Military Medical Center

**Site Principal Investigator:**

David Morgenroth, MD, VA Puget Sound Health Care System

**Collaborators:**

Overall Project PI: Bradford Hendershot, PhD, Walter Reed National Military Medical Center (WRNMMC)

Benjamin Shuman, PhD, Seattle Institute for Biomedical and Clinical Research (SIBCR)

# Introduction

**Burden of injury.**

Over 17,200 U.S. Service members have been medically evacuated from theater due to battle or musculoskeletal-related injuries sustained during Operations Iraqi Freedom (OIF), New Dawn (OND), and Enduring Freedom (OEF) [1]. Global military figures show that the largest burden of injury from the global war on terrorism is extremity trauma [2], which represents 64% of the $170-million projected disability benefit costs and causes the largest percentage of days on limited duty [3]. Nearly 50% of all extremity injuries involve the lower extremities [4], thus impacting mobility, community participation, and the overall quality of life of affected individuals. With the advancement of forward medical care on the battlefield, there is a corresponding increase in Service members surviving with these major lower extremity traumatic injuries which previously would have resulted in amputation. The military has a vested interest in maximizing injured Service member function and quality of life across the continuum of care. The relatively young mean age of these Service members implies higher vocational and recreational demands and expectations.

**Ankle-foot orthoses (AFOs).**

AFOs are often necessary to overcome mobility limitations related to lower limb neuromusculoskeletal injury. The availability of orthotic options for improving function is expanding, but still limited [5]. Relative to the field of prosthetics, orthotics research and development has remained relatively understudied and underfunded for decades. Moreover, outcomes are better for individuals who have sustained lower limb amputation than for those who have had their limb salvaged or reconstructed [6], which is potentially due to specialized technology available and provided to those with amputation, and not to those with limb salvage [6]. To date, a wealth of information on comparative effectiveness of designs, standardization of certain outcome measures, and surveys exist to identify the needs and preferences of prosthesis users, but the same level of scientific evidence does not exist for AFO users. The current evidence is insufficient to develop Clinical Practice Guidelines. Despite the lack of scientific evidence, Medicare expenditures for orthotics in 2016 reached $1 billion [7]. A 2015 National Commission on Orthotic and Prosthetic Education (NCOPE) study projected that by the year 2025, the number of certified orthotists will need to increase by 90% to meet future patient demands [8]. There is a critical need to better optimize orthotic devices for patients and provide evidence-based tools for clinicians. The proposed study with its unique test-drive strategy will address these unmet needs by optimizing the process of selecting an AFO in a patient-centered and patient-specific manner.

**Limitations of available information to guide AFO prescription.**

The scarcity of publicly available objective orthotic mechanical property (e.g., stiffness) data and different vendor stiffness naming paradigms (e.g., numbered 1-7, arbitrary units of measurement, color names) limits clinicians’ ability to choose an AFO that optimally matches a given patient’s needs. It is a constant challenge to compare devices across vendors to hone in on the ideal design features for a given patient.

The interaction between the user and the AFO is largely determined by its mechanical properties that can impact the degree to which movement is either restricted or augmented. One key AFO design mechanical property is stiffness. The stiffness of the device about the ankle impacts the extent to which the device offers energy storage and push-off enhancement during waking. For example, a stiffer device offers less range of motion but may provide greater support and higher rates of energy storage and return, which may be desirable for someone wishing to engage in higher impact activities. There is evidence to support that an optimal match exists between an individual’s gait-related impairments and AFO ankle stiffness [9]. There is also an optimal stiffness at which energy expenditure during walking can be reduced [10-13]. Thus, optimizing AFO stiffness is an important aspect of optimally restoring mobility and participation in activities of daily living in individuals with lower limb deficits resulting from musculoskeletal trauma. However, there is limited evidence to guide this process. Quantitative stiffness data, for example, are only available for select devices in the research literature, often only comparing a few devices, many of which are custom fabricated orthoses. A recent review paper by Totah et al. [14] summarized stiffness values about the ankle joint from a range of devices, often having to estimate values from data provided. This lack of objective mechanical properties data and lack of standardization underscores the need for a test-drive strategy for AFO prescription.

**Current prescription process.**

Optimal AFO prescription should align the needs and abilities of the patient with the functional characteristics of the device but, once again, evidence is limited. Currently, the AFO prescription process relies on clinician intuition, training, experience, and qualitative guides provided by vendors. Clinical evaluation for AFO prescription is typically based on patient’s physical examination (e.g. muscle strength and joint ranges of motion), functional evaluation, medical history, body mass, and overall mobility goals. The clinician then selects from the commercially available devices, of which there are a number of makes and models of varying materials, designs, and componentry. For instance, mechanical joints can permit range of motion while stops can be incorporated to limit motion. AFOs may incorporate a variety of materials in design: thermoplastics, composites such as fiberglass or carbon fiber and resin, metals, and leather. Each material, and the way in which it is integrated into the device, imparts different mechanical properties. AFOs may be prefabricated and available off the- shelf or custom molded to the patient. Custom carbon fiber dynamic AFOs are now being used more frequently. These designs have been developed to offer energy-storing-and returning (ESR) properties and are commonly intended for patients who desire to participate in high impact activities. However, matching the right device with the patient can be challenging. Moreover, to reduce expense and time, clinicians are typically tasked with getting it right on the first try. This is a significant limitation in clinical care to which our proposed test-drive strategy (described below) will address.

**AFO user input in the prescription process.**

Successful patient adoption of an AFO device relies on patient satisfaction and perceived mobility, thus there is substantial benefit to incorporating patient feedback into the AFO prescription process. Moreover, patients using lower limb assistive technology routinely want and expect their input to be considered in their plan of care [15, 16]. Involving the patient in the clinical decision making process has been shown to improve functional outcomes [17]. However, there are limited opportunities for patients to try out different devices and offer feedback, particularly on custom-made AFOs. Trial and error of numerous devices (particularly for custom devices) would be inefficient, expensive, and highly labor-intensive. Therefore, patients are unable to explore the range of options the way they would be able to with footwear in a shoe store. In a shoe store, a customer may try any number of shoes within a given category (walking or running, for example) that may meet his or her needs. An AFO user’s only experiential comparison is often between the new selected device and not having any prior device or between the new device and a previous device that the patient had identified as not well-suited for his or her needs.

Numerous comparative effectiveness studies have sought to identify the best device for a patient. However, the post-hoc tests are generally only capable of identifying the better device between two. Determining which AFO provides optimal patient outcomes is a challenging task. There is no singular optimization criterion that will work for all patients. Thus, we propose a test-drive strategy for different AFO devices focused on improving satisfaction, function and mobility. This novel strategy has great potential to streamline the prescription process, enable the patient to provide experiential feedback, and improve outcomes.

**Test-drive strategies.**

To address the limitations in the current prescription process, we have developed a commercially available, customizable robotic exoskeleton emulator (Caplex system, Humotech, Pittsburgh, PA) that is worn around the ankle joint. The system includes the off-board control system and actuator unit, the ankle-foot exoskeletal end effector (worn by the user), shoes that can be quickly swapped with the device, and the flexible tether. The exoskeleton is actuated and controlled by offboard actuator unit and control hardware. Thus, this setup delivers an ultra-powerful exoskeleton end-effector with minimum worn mass and inertia. The wearable component only weighs 1.4 kg (including the mass of an athletic shoe). The exoskeleton is capable of emulating active or passive device conditions by applying torque based on time or ankle angle, depending on different applications. Benchtop experiments have demonstrated the emulator’s exceptionally high torque control performance [18-20]. An impedance control law can be used to mimic the elastic behavior. This system has successfully been used to emulate a broad range of passive, spring-like properties [20], making it well suited for applications with passive-dynamic AFOs. Thus, the system can be programmed to emulate the behavior of a broad array of commercially available (*actual*) AFOs without requiring the user to physically change devices. This provides the wearer with the experience of swapping out different design features in real time via software interface without the costly and time-intensive trial and error process of comparing *actual* devices. The emulator is not meant to replace clinical expertise but to augment the tools currently available to better optimize the design and prescription of AFOs.

# Objectives

The overarching goal of this research is to optimize mobility, function and satisfaction for Service members, Veteran, and civilian AFO users with lower limb musculoskeletal injuries. To achieve this, our primary purpose is to determine the extent to which a ‘test-drive’ strategy, using an AFO emulator, can be used to predict device function, mobility and preference outcomes with corresponding commercially available (*actual*) AFOs in Service members and Veterans with lower limb musculoskeletal trauma.

**Specific Aim 1**: Test the ability of the AFO emulator to reproduce the user experience of wearing *actual* AFOs during different in-lab activities (walking, running, stair climbing; level ground, ramps) in individuals with lower limb musculoskeletal injury. This aim will determine if the proposed AFO emulator framework can provide an option for quickly exploring multiple devices in a short period of time.

**Research Question 1a**: Do self-reported measures of preference differ between *emulated* and corresponding *actual* AFO conditions?

**Hypothesis 1a**: Preference scores for *emulated* AFO conditions will be strongly correlated with preference for the corresponding *actual* AFO.

**Research Question 1b**: Do measures of performance and self-reported outcomes differ between *emulated* and *actual* AFOs conditions?

**Hypothesis 1b**: Measures of performance and self-reported outcomes in the *emulated* AFO condition will be strongly correlated with those using corresponding *actual* AFOs.

**Specific Aim 2*:*** Test the ability of a short-term, in-laboratory evaluation of function, mobility, and preference to predict those same outcomes longer-term (i.e., after 2 week use in the community) in AFO users with lower limb musculoskeletal injury. This aim will determine the predictive ability of a brief test-drive strategy using *actual* and *emulated* AFOs to identify function, mobility, satisfaction, and preference at follow-up after two weeks of use in the community environment with corresponding *actual* AFOs.

**Research Question 2a**: Do measures of user preferences identified during a brief test-drive session with an *emulated* AFO predict user preferences after the *actual* AFO is worn longer-term in the community environment?

**Hypothesis 2a**: Initial preference scores for each *emulated* AFO will be strongly correlated with preference for the corresponding *actual* AFO at follow-up.

**Research Question 2b**: Do measures of user preferences identified during a brief test-drive session with an *actual* AFO predict user preferences after the same *actual* AFO is worn longer-term in the community environment?

**Hypothesis 2b**: Initial preference scores for each *actual* AFO be strongly correlated with preference at follow-up.

**Research Question 2c**: Do measures of user preferences identified during a brief test-drive session correlate with performance measures and self-reported measures of mobility after the same *actual* AFO is worn longer-term in the community environment?

**Hypothesis 2c**: Initial *emulated* preference scores be strongly correlated with performance-based and self-reported mobility measures at follow-up.

**Research Question 2d**: Do measures of user preferences identified during a trial with the *actual* AFO correlate with performance measures and self-reported measures of mobility after the same AFO is worn longer-term in the community environment?

**Hypothesis 2d**: Initial *a*ctual preference scores be strongly correlated with performance-based and self-reported mobility measures at follow-up.

**Exploratory Aim:** Allow the user to self-tune AFO emulator stiffness settings while walking (***patient-optimized***) and compare function, mobility, preference to the clinically prescribed AFO (***clinician-optimized***). This aim will determine if an AFO test drive strategy can be used to better optimize user satisfaction and patient reported outcomes relative to the current standard of care.

**Research Question 3a**: How does preference differ between the patient-optimized setting on the AFO emulator and in the clinically-prescribed *actual* AFO?

**Hypothesis 3a**: Preference scores for the user-optimized setting on the emulator will be greater than those in the clinically prescribed AFO.

# Study Procedures

## 4.1 Study Design

This is a clinical trial with a participant-blinded, cross-over study design with repeated-measures assessments will take place at each site. Short-term, in-lab sessions will be used to address Aim 1 (i.e., comparing in-lab *emulator* trials to in-lab corresponding *actual* AFO trials) and the Exploratory Aim (comparing in-lab patient-optimized emulator trials to in-lab prescribed *actual* AFO trials). Aim 2 will compare short-term in-lab trials with longer-term follow-up using corresponding *actual* AFOs. We will assess user preference, satisfaction, perceived mobility and performance in *emulated* and *actual* AFOs to determine whether the emulator and/or a brief trial of *actual* AFOs can predict user mobility and AFO preference following longer term use in the community environment.

See section 5.5 below for data collection procedures.

## 4.2 Recruitment Methods

The study will recruit up to 56 participants with lower limb musculoskeletal trauma who are currently using an AFO. Men and women will both be recruited to the extent they are represented in the patient population. No exclusions will be made on the basis of race, ethnicity, sex, or gender.

Human subjects will be recruited from a convenience sample of eligible participants at the sites. The sites were specifically selected based on their potential for high enrollment and because they prescribe a wide range of AFO devices to patients of various mobility/activity levels and injury etiologies.

The following methods and resources may be used to recruit subjects:

1. Recruitment using medical record data is often believed to be the most effective means of achieving enrollment goals [21]. Designated research staff at the sites will screen relevant clinic lists in computerized patient record systems to identify potential participants. After review of relevant clinic lists, designated staff will go to the clinic or contact providers to ask if a patient might be a good fit for the study. If the clinician agrees that a patient may be an appropriate study participant, during an appointment the clinician will ask the patient if she/he is interested in speaking with designated study staff; patients will be given a chance to opt out. For patients who are interested, designated study staff will speak to potential participants.

2. **Clinician Referral:** The study teams at each site have built relationships with local clinic staff and have established effective clinical referral systems. Research staff will inform providers working in relevant clinics about the study and inclusion/exclusion criteria so they can refer potential participants to contact the study team. Flyers and business cards may be provided to clinicians to give to patients that are interested in the study. If a clinician informs patients about the study and the patient agrees to be contacted about it, the clinician may provide the research team with the patient’s name and they may also provide contact information. Clinicians may also provide (via in-person/on the phone) the names of patients that they are aware of who may be a good fit for the study and a study staff member will send an approach letter, make direct contact during a clinic visit, or the clinician provider may inform potential participants.

3. **Flyers:** Approved flyers may be posted in designated areas at the sites. Flyers will also be posted and distributed to potential subjects at various locations. Permission will be obtained prior to posting flyers in these clinics. Clinicians and support staff will direct interested individuals to contact the study staff to learn more about the study. The flyers may also be re-sized to be used in print publications or as a complete image in online ads. QR codes may be used on flyers so participants can take a picture and then a pre- populated email can be sent to the research team to identify an individual’s interest in a particular study. Recruitment advertisements may also be in print and online publications, as permitted by the IRB.

4. **Participant Registries:** Applicable patient registries maybe also be used for recruitment. The registries would also include participants that were screened and/or participated in previous studies and includes lower limb prosthetics and orthotics users. Designated study staff may also speak with these potential participants in-person if they have an upcoming clinic visit.

Payment of Participants

Each participating site may advertise via different recruitment efforts. Participants may be paid up to $500 total for study participation by HJF. Payment will be dispensed after completing each research milestone. There is no cost to patients for the research tests, procedures/evaluations, and study product while taking part in this trial. Procedures and treatment for routine clinical care not directly associated with the study including costs associated with hospital stay may be billed to the patient, patient’s insurance, or third party.

| Payment Schedule | | |
| --- | --- | --- |
| Study Visit 1 | Study introduction, screening, consent, casting | $50 |
| Fittings | AFO fittings (if required, 1-2 sessions) | $30 each |
| Study Visits 2-3 | In-lab testing sessions | $80 each |
| Study Visits 4-6 | In-Lab testing sessions following 2-week trials | $50 each |
| Study Visits 7 | In-lab exploratory testing session (optional) | $80 |
| Total |  | $360-500 |

## 4.3 Informed Consent Procedures

A waiver of informed consent and HIPAA authorization will be used for initial recruitment and screening purposes. A waiver of documentation of consent and HIPAA authorization will be used to retain the preliminary eligibility screening responses. Informed consent will be obtained prior to enrollment in the study. A member of the research team will conduct the informed consent process and screening based on the inclusion and exclusion criteria above.

## 4.4 Inclusion/Exclusion Criteria

**Inclusion Criteria**

- lower extremity injury requiring current use of an AFO
- unlimited community ambulators [22]
- age 18-65 years
- 100-250 lbs
- at least 2 weeks’ experience using an AFO
- ankle/foot pain over the last week on the foot which you wear an AFO, asked at screening: average resting pain <1/5; average pain with activity <2/5
- U.S. Men’s foot size 7-13
- >10 degrees sagittal plane ankle range of motion
- English speaking
- able to comply with study procedures

**Exclusion Criteria**

- known cognitive impairment (e.g. diagnoses such as moderate/severe traumatic brain injury, dementia)
- inability to comply with instructions
- medical conditions that would preclude safe involvement
- unable to provide written informed consent
- pregnant (as per self-report)
- prior history of stroke
- use of an assistive device such as a walker or cane

## 4.5 Study Evaluations

Prior to enrolling and consenting, participants must meet the inclusion criteria described above. Once consented, AFOs will be fit to each subject at an initial visit, based on manufacturer recommendations for foot size and body weight. Additional fitting sessions may be utilized to ensure a good fit to the individual for all three actual AFOs to be evaluated.

Participants will undergo up to three in-lab sessions: testing the emulated AFOs, testing the actual AFOs, and an optional exploratory aim session of user-selected AFO properties. Participants will undergo three 2-week periods where participants will be instructed to wear the study AFO instead of their clinically-prescribed AFO for all usual activities of daily living. After two weeks of wearing each AFO, participants will return to the laboratory for follow-up evaluation (Figure 1). Injury history and participant demographics will be collected at the initial intake session.

Participants will be block randomized to ensure similar numbers of participants are assigned to each of three AFO testing sequences and similar numbers of participants undergo either the emulated in-lab session or actual AFO in-lab session first.

**Emulated AFO testing session**. Participants will begin by walking on a treadmill in the emulator to become accustomed to its use. Participants will wear the emulator’s end effector the way they would their clinically prescribed AFO and the study orthotist will be present to make any necessary adjustments. The emulator parameters will be adjusted by the research staff member in real-time as the subject walks so they experience the range of emulated AFO conditions to be tested. After the emulator accommodation period, participants will trial three emulated AFOs in a randomized order. Participants will complete an Activities Trial and patient reported outcomes (described below).

**Actual AFO testing session.** Prior to the start of the testing session, participants will be fit with the three actual AFOs to be worn during the data collection. Fitting will be done according to the routine standard of clinical care (e.g. test device for the custom AFOs). On the day participants complete the actual AFO testing, they will walk on the treadmill in each AFO condition to acclimate prior to testing. Then, participants will undergo the same Activities Trial as in the emulated AFO testing session (described below). The same questionnaires will be completed to identify patient-reported outcomes. In the actual AFO testing session, participants will additionally complete a series of performance tests described below.

**Two-week follow up sessions**. Participants will complete two-week long community trials in each of the three actual AFO conditions in a randomized. AFOs will have already been fit to each subject as described above. Participants will be instructed to wear the study AFO instead of their clinically-prescribed AFO for all usual activities of daily living. After two weeks of wearing each AFO, participants will return to the laboratory for follow-up evaluation; Patient-Reported Outcomes and Performance Tests will be assessed. A subset of participants may also have the activities trial collected and/or gait biomechanics collected at the two-week follow up sessions.

**The Exploratory Session** will compare a participant’s clinically prescribed AFO (clinician optimized) to a participant-optimized setting on the emulator. Stiffness will be the optimized parameter. In this exploration, participants will be allowed to dial in and change the stiffness parameters on the emulator so that they self-identify an optimal value (patient-optimized).

**Figure 1.** Study Overview

**
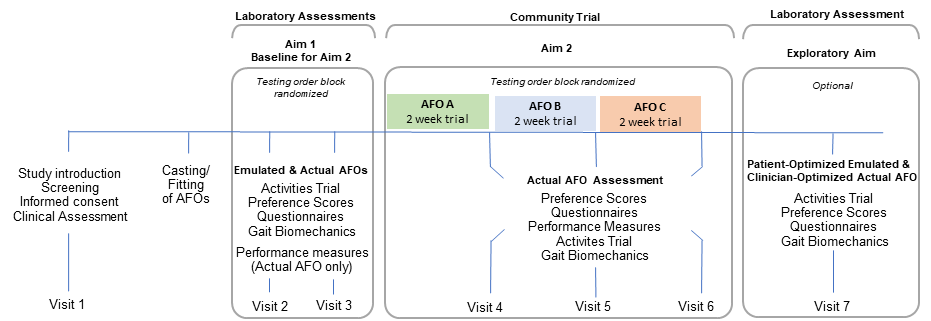
**

**Activities Trial**. The activities selected are intended to represent a range of activities commonly encountered in the community environment (Table 1). As detailed below, activities will be assigned in a manner specific and appropriate to the individual patient (e.g., participants of a lower mobility or with contraindications will not be asked to run). Each speed and activity condition will take place for at least 30-seconds and rest will be provided between activities as needed. After each AFO condition is complete, participants will rest for at least 10 minutes before engaging in the next AFO condition.

| **Activities Trial** |
| --- |
| Walking on a level treadmill at 3 standardized speeds based on leg length [23] |
| Walking uphill on a treadmill at a comfortable speed |
| Walking downhill on a treadmill at a comfortable speed |
| Walking up a stairmill at a comfortable pace (optional) |
| Running on a treadmill at a comfortable speed (optional) |

**Table 1.** Activities trial conditions

During the activities trials we will collect gait biomechanics. Participants will be asked to change into a tight-fitting shirt and shorts that we can provide. A private area for participants to change clothes will be provided. We will record a series of standard body measurements such as height, weight, and lengths of segments. We will attach up to 70 reflective makers using double sided tape and wrap to the participant’s body segments and joints. During the tests in the lab, the cameras will record the location and movement of the markers; they do not record a picture of the body. Ground reaction force data will be collected from an instrumented treadmill during treadmill walking.

**Patient-Reported Outcomes.** Participants will complete a series of questionnaires to assess patient-reported outcomes. Some of the questions in these instruments are not sensitive to a short-term trial and may be excluded or modified (see descriptions below).

• **AFO Preference**. Preference is the primary outcome measure. Preference for a given condition will be assessed using a 10-point scale ranging from “Do not like at all” to “Like very much”. Each participant will be asked to rate his or her preference during each specific trialed activity. During in-lab testing, preference will be assessed at the conclusion of the Activities Trial. Additional information of the user’s assessment of the AFO stiffness ranging from “Far too stiff” to “Far too soft” and whether they would wear the device on a daily basis will be collected.

• **AFO Comparison**. Ranked preference and perceived AFO stiffness will be collected at the actual and emulated in-lab session between the three braces trialed and following the third community take home trial.

• **Rating of Perceived Exertion** (RPE). RPE will be recorded on a CR 10 Borg scale [24, 25]. RPE will be recorded after each activity in the Activities Trial and after the 2-Minute Walk Test (see below).

• **Pain**. Ankle/foot pain on the limb with the AFO will also be scored using on an 5-point scale (1 - no pain to 5 – very severe). Pain will be recorded after each activity in the Activities Trial and after the 2-minute-walk-test (see below). During the community trial phase, pain will be assessed at the conclusion of each two-week AFO trial period. Pain will also be assessed as a screening measure (see inclusion/exclusion criteria). using the PROMIS Pain Intensity (short form 3a) and Interference subscale (short form 6b). During the community trial phase, pain intensity and interference will reflect the past seven days. The PROMIS Pain Intensity and Pain Interference subscales are universal, rather than disease specific, have high reliability, and high construct validity with related health outcomes [26].

• **Activities-Specific Balance Confidence Scale** (ABC-5 Scale). The ABC-5 Scale is a subjective measure of confidence in balance or unsteadiness when performing a variety of movements. These movements are specific to activities of daily living. It can be used to assess fall risk among cohorts of individuals with balance limitations. For example, scores of <67% indicate a risk for falling and can accurately classify individuals who fall 84% of the time [27]. This scale has been used in a wide variety of patient populations with lower limb impairments, including individuals with lower limb trauma, such as amputation [28, 29]. The ABC will be assessed at intake to characterize the participant cohort. The questions relating to walking around the house (substitute “treadmill”), walking up stairs, and walking up and down a ramp will be used for in-lab testing and the ABC will be assessed at the conclusion of the Activities Trial in each AFO condition. During the community trial phase, the full ABC will be assessed at the conclusion of each two-week AFO trial period.

• **Quebec User Evaluation of Satisfaction with assistive Technology** (QUEST 2.0). The QUEST 2.0 evaluates a user’s satisfaction with various assistive technologies [30]. This 12-item instrument incorporates eight items to assess characteristics in terms of 1) size, 2) weight, 3) adjustments, 4) safety, 5) durability, 6) simplicity of use, 7) comfort, and 8) effectiveness. Subjects are asked to rate their satisfaction for the device on a five point scale. Four additional items related to service include 9) service delivery, 10) repairs and service of the device, 11) professionalism of service, 12) follow-up service will not be assessed as part of this study. Subjects are asked to choose the three most important items related to their assistive device. The QUEST 2.0 has been used in a variety of patient populations with lower limb injuries, including individuals with amputation [31] and in a range of orthotic applications [32]. The QUEST will be assessed at the start of the initial session to characterize the participant cohort. The QUEST will also be assessed at the conclusion of each two-week AFO trial period.

• **Orthotics and Prosthetics Users’ Survey** (OPUS). The OPUS is a self-report questionnaire consisting of five modules [33]. In the proposed work, we will be incorporating the following two modules related to the lower extremity: 1) Lower Extremity Functional Status Measure and 2) OPUS- Satisfaction with Devices and Services (questions 1-11 only). The first is based on a five-point Likert scale and nominal Yes/No scale while the second uses a six-point Likert scale. The OPUS can be used in all ages and with unilateral or bilateral prosthetic/orthotic users, congenital or acquired populations, and with all levels of orthosis and/or prosthesis use. The OPUS will be assessed at the start of the initial session to characterize the participant cohort. During the in-lab testing, the OPUS Lower Extremity Functional Status Measure questions relating to balance while standing, walking around indoors, walking up a ramp, and climbing a flight of stairs will be used. All questions will be assessed following each community trial test All questions relating to satisfaction with device (1-11) will be used after each community trial test.

• **Orthotic Patient Reported Outcomes – Mobility** (OPRO-M). The OPRO-M is a self-report 20 item questionnaire (publications in preparation) examining the ability of the participant to complete a range of challenging everyday mobility tasks with their assistive device(s). The OPRO-M is based on a five-point Likert scale. The OPRO-M will be assessed at the start of the initial session to characterize the participant cohort and following each community trial test.

• **Self-Reported Wear Time.** The participant will be asked to record approximately how many hour the study brace/AFO was worn during each day of the take home period.

• **Feedback.** The participant will be asked to provide any feedback they may have on the study, their participation in the study, or the braces used at the conclusion of their participation in the study.

**Performance Tests**. Participants will complete a series of performance tests. These measures each relate to the questionnaire-based measures described above. For example, the Narrowing Beam Walking Test objectively measures balance while the ABC subjectively infers balance from self-report.

• **Two-Minute Walk Test** (2MWT). The 2MWT assesses walking ability. Participants will be asked to walk the greatest distance they can in 2 minutes while walking a 15.2m ‘out and back’ course until asked to stop [34]. The distance walked will be recorded as will the post-test rate of perceived exertion (Borg CR 10 scale) [35]. The 2MWT has been routinely as part of prosthetics and orthotics research. The 2MWT has been found to be valid and reliable in multiple diagnostic groups including those with lower limb amputation, neurologic impairment and others [36]. Meta-analyses and reviews report improvements in walking and mobility as a result of AFO use, so it is reasonable to expect that improvements in these areas [37-39].

• **Four-Square-Step-Test** (FSST). The FSST is a test of balance and agility [40] where the patient must change directions quickly while maintaining balance. A 1-inch high obstacle in the shape of a Maltese cross is placed on the floor. The participant is instructed to begin in the back, left quadrant then to move 1) forward, 2) sideways right, 3) backward, then 4) sideways left, then to move in the reverse direction back to the original square. They move as fast as possible through their movement progression while keeping one foot on the floor at all times. The only materials required are pieces of PVC pipe and a stop watch. Participants will complete one practice and two successful trials. In healthy, active duty Service members, inter-rater reliability was excellent (ICC = 0.99, 95% CI = 0.99-0.99) and test-retest reliability was good (ICC = 0.86, 95% CI = 0.75-0.96) [41]. In individuals with amputations, the Four-Square-Step-Test has high sensitivity (92%), specificity (93%) and predictive values (positive: 86%; negative: 96%) for identifying fall risk.

• **Narrowing Beam Walking Test** (NBWT). The NBWT is a reliable and valid task that challenges balance abilities in healthy and impaired populations [42, 43]. Participants walk with arms crossed along a 6.71m beam of narrowing width until they can no longer maintain balance and step off the beam. The distance of the last foot position on the beam is recorded. Five trials can accurately estimate usual balance performance in groups such as lower limb prosthesis users [43]. The NBWT demonstrates strong correlations (0.70-0.85) with performance on other balance tasks such as TUG, FSST, and Berg Balance Scale and moderate correlations (0.49) with the ABC Scale. Performance is significantly lower among individuals of lower mobility levels. There are no floor or ceiling effects indicating that the NBWT measures broad ranging balance abilities without reaching scale limits [42].

• **20-Meter Shuttle Run**. The 20-Meter Shuttle Run has been used in other ESR AFO research [44]. It is a non-standardized measure intended to assess power, deceleration, and ability to change pace [43]. It is used in this study for its resistance to ceiling effects, particularly for high-activity ESR AFO users. Participants will be instructed to move to a cone 10 meters away and then return to the starting point as fast as possible. Each subject will be asked to complete three trials at each point of testing, with up to five minutes of rest between trials. The fastest of the recorded times will be used to score each instance of testing. Participants that are not able to run will be asked to walk as quickly as is safely possible to complete the course and may skip the assessment if otherwise not able to complete.

**Photos and Video** We may take video and photos of participants during portions of this study for documentation and use in research publications. To protect the identity and privacy of our participants, all videos and photos will be edited later to de-identify the images (e.g., blurring of faces, tattoos, and other distinguishing marks). No sound will be recorded to prevent voice identification.

**Additional Use of Coded Data** Throughout the study we may place copies of all coded data in publicly accessible online repositories. Once posted, the coded data will be publicly accessible to search, retrieve, and analyze for any purpose. Participants will be made aware of this use of coded data during the consent process and it will be descried in the consent form. If participants do not wish to have their coded data placed in online repositories they may choose to not participate in the study. We will also share coded data between the research team members at WRNMMC, VAPSHCS, SIBCR, and HJF.

***Potential Risks to Participants*** There is a risk of injury or physical discomfort associated with the device casting, fitting, wear, or testing during study participation. Wearing the emulated AFO may also cause discomfort. There is always the risk that the emulator could fail or stop working properly. This could cause minor soft tissue injury (i.e. soreness, bruising) or other minor injury (i.e. knee or ankle joint soreness). There are built-in emergency stops designed to prevent injury and Humotech currently has 15 emulator systems in use with no reported safety concerns. The risks of the emulator are similar to those of wearing the actual AFO. These risks are considered minimal risks since the activities are consistent with those performed during physical therapy evaluations or AFO fittings, and the devices can be removed at the participant’s discretion. Device fittings will be overseen by a certified prosthetist-orthotist and data collection will be conducted by trained research staff. Furthermore, if a participant cannot safely perform a test once begun, the test will be stopped and the participant will proceed to the next test or try again. If the participants experience any pain or discomfort during or after testing, they will be instructed let the research staff know. Participants may experience fatigue during the assessment sessions. If fatigued, participants will be instructed inform research staff and will be allowed to rest until they are comfortable or decide to discontinue.

There is always the possibility that participants may fall while walking in an AFO but procedures have been put in place to minimize this risk. All study interventions are non-invasive. There may, however, be unforeseen risks associated with this study.

Some of the questionnaires may make participants feel uncomfortable or embarrassed. This is not expected based on the nature of the study, but if they experience these emotions they can decline to answer the questions.

Although we make every effort to keep participants information secret, there is always a chance of loss of confidentiality through someone gaining access to the information that researchers have collected. Section 16 outlines how we will protect your privacy to the best of our ability.

***Potential Benefits to Participants***

There are no direct benefits to participants for taking part in the study. However, others may benefit in the future from the information learned during this study. The possible benefits to others are creating a better process for determining which AFO is best to prescribe to a patient. Participants will be compensated for their time.

## 4.6 Data Analysis

**Aim 1.** The purpose of Aim 1 is to test the ability of the AFO emulator to reproduce the user experience of wearing actual AFOs during different activities (walking, running, stair climbing; level ground, ramps) in individuals with lower limb musculoskeletal injury. We will consider the outcome measures as continuous response variables in the statistical analysis. We will evaluate the data from the in-lab actual and in-lab *emulated* sessions using a linear mixed effect (LME) regression with the *emulated* AFO data as the independent fixed effect, the *actual* AFO data as the dependent fixed effect, and subject participant as a random intercept.

**Hypothesis 1A**. In Specific Aim 1A, we are testing the hypothesis that the experimental AFO preference measured during the in-lab *emulated* session will be significantly correlated with the experimental AFO preference measured during the in-lab *actual* session. This model estimates a change in *actual* AFO preference per unit change of *emulated* AFO preference.

**Hypothesis 1B**. In Specific Aim 1B, we are testing the hypothesis that self-reported measures and biomechanics will be significantly correlated between the in-lab *emulated* session and the in-lab *actual* session. This model operates the same as in 1A, substituting measures of function and mobility for preference.

**Aim 2.** The goal of Aim 2 is to determine if the in-lab (*emulated* and *actual*) measurements are a good predictor of the longer-term measurements made following a take home trial. To perform this analysis, we compute a similar LME regression as Aim 1 with the *emulated* *or actual* in-lab AFO data as the independent fixed effect, the *actual* take-home trial AFO data as the dependent fixed effect and subject participant as a random intercept.

**Hypothesis 2A**. In Specific Aim 2A, we are testing the hypothesis that the experimental AFO preference measured during the in-lab *emulated* session will be significantly correlated with the experimental AFO preference measured during the take home-trials. This model operates the same as in 1A, substituting measures of take-home trial preference as the dependent variable.

**Hypothesis 2B**. In Specific Aim 2B, we are testing the hypothesis that the experimental AFO preference measured during the in-lab *actual* AFO session will be significantly correlated with the experimental AFO preference measured during the take home-trials. This model operates the same as in 1A, substituting measures of take-home trial preference as the dependent variable.

**Hypothesis 2C**. In Specific Aim 2C, we are testing the hypothesis that measures of preference will be significantly correlated between the *emulated* in-lab session and measures of performance and self-reported measures of mobility at the take-home trials. This model operates the same as in 2A, substituting measures of performance and self-reported outcomes for preference as the dependent variable.

**Hypothesis 2D**. In Specific Aim 2D, we are testing the hypothesis that measures of preference will be significantly correlated between the *actual* in-lab session and measures of performance and self-reported measures of mobility at the take-home trials. This model operates the same as in 2A, substituting measures of performance and self-reported outcomes for preference as the dependent variable.

**Power Analysis for Aims 1 & 2**. Our power analysis is based upon pilot data collected for a similar study using prosthetic feet which tested mobility and preference for three participants and two feet between *emulated* and *actual* sessions. Based upon the estimates of slope and residual error from the pilot data, 10,000 datasets were created for a sample size of N=50. Power, estimated as the proportion of datasets that rejected the null hypothesis of a slope equal to zero, was 97% for the preference scores and 98% for the mobility scores. To account for a 12% dropout rate (note that prior prosthetic emulator study experienced a 7% dropout rate) a total sample of N=56 participants will be tested.

**Exploratory Aim**. In this exploration, participants will be allowed to dynamically “dial-in” and change in the stiffness settings of the emulator device so that they design an optimal device to meet their needs. The *emulated* device will then be compared to the device that was clinically prescribed for them as part of usual care. To test the hypothesis a simple paired t-test will be used which compares the preference means and other outcomes measures of the patient-optimized and clinician-optimized conditions.

## 4.7 Withdrawal of Subjects

A study clinician or PI may withdraw a participant without their consent if he or she feels that it is not in a participant’s best interest to continue in the study or the person is unable to complete the study procedures. All data previously collected from participants who withdraw or are withdrawn will be kept and may be used in the study data analysis. Participants may withdraw at any time by informing the Research Coordinator and/or the PI.

# Reporting

All safety information on AEs, SAEs, unanticipated events or problems, and protocol deviations will be collected. This information will be collected at study visits and whenever subjects call to report a problem. The information will be collected on VA IRB forms (Report of a SAE and/or Problem Form, or Report of Problems (ROP) Form) and in AE log forms as needed. Safety data will be collected on an as-needed basis and will begin upon enrollment into the study. All safety reporting requirements will be followed. Any anticipated AEs will be recorded on a log sheet and reported annually with the CRQ.

Unanticipated SAEs, protocol deviations and/or serious problems that are related to the

research will be reported to the Central IRB within 5 business days of becoming aware of the event. After each report of a U-SAE or problem, and/or SAE, the Medical Monitor will evaluate study procedures for previously assessed risks, and will determine whether any changes must be made to minimize risks. The Medical Monitor has the authority to stop the research protocol in progress, remove individual participants from the research protocol, and take whatever steps are necessary to protect the safety and well-being of human subjects until the IRB assesses SAEs or other reports. Reports for events determined by either the investigator or Medical Monitor to be possibly or definitely related to participation and reports of events resulting in death will be promptly forwarded to the USAMRMC ORP HRPO. Cumulative safety data will be reviewed yearly by the Site PIs and the Medical Monitor, and will be reported to the VA and DoD IRBs during continuing reviews. The above procedures are standard practice at VAPSHCS and WRNMMC and we will involve a Medical Monitors with whom we have worked previously in this capacity.

# Privacy and Confidentiality

As with any study, it is possible, although unlikely given the impersonal nature of the data collected, that participants may experience a loss or invasion of privacy or confidentiality because of participation in this study. The risk of harm is minimal and the protections described here will be followed.

Participant data will be collected the study sites and will be entered into independent and secure databases. In general, electronic data will be coded with links stored separately, stored on password protected drives, servers will be backed up regularly, firewalls in place, and access permitted only by study team members listed on the approved IRB protocol. Hard copies of data forms will contain only codes and be stored in locked cabinets (dedicated to this study’s records), and locked offices with access only given to study team members. Informed consent documents will be stored separately along with other identifiable information (such as records necessary to have in paper form, which will be limited). Because this is a multi-site study, extra care will be taken not to share PHI or other identifiable information about participants across centers.

To protect privacy and confidentiality during data collections, all data collected will have PHI removed and participants will be issued an alpha-numeric code which will be used to track their records. Subject records will be coded with a unique identifier. The first five characters will be an abbreviation of the study (AFO Test Drive - AFOTD) to identify to which study the subject belongs followed by a site identifier (WR = Walter Reed, VA = VAPSHCS) and sequential numbering based on the subject order of enrollment. The device tested will be indicated as device 1, 2, or 3.

# Communication Plan

All human subjects data collected as part of this study will be handled in a manner compliant with the intent and letter of the regulatory processes at each of the site. Data will be collected locally at each of the sites and initial analysis will take place at each site. No PHI or PII will be shared. Only coded data will be shared across sites. The investigators agree to adhere to any and all local policy guidelines for data sharing.

1. **Information Security and Data Storage/Movement**

Non-sensitive electronic data labeled with the study assigned codes and all 18 HIPAA identifiers removed (coded data), may be stored on password protected equipment (computers/laptops/hard drives) but will not be encrypted.

To minimize the risk of the loss of confidentiality and provide for data security, conservative data management procedures have been established at all study locations involved in this project. To facilitate successful completion of the Specific Aims, identifiable data will be shared only internally. Chart reviews of coded data will be completed locally and only coded human subjects data will be shared among the sites via file transfer software, email, and/or other electronic media (CD/DVD/USB drive). Throughout the course of the study, we may place a copy of all coded data in publicly accessible online data repositories. Additionally, we intend to make our datasets publicly available at the conclusion of the study in publications and presentations. We will also share our mechanical testing (non-human subjects) results with Humotech, the emulator vendor so they can program the system. Therefore, there are no anticipated concerns with Humotech (or others) incorporating this data as part of repository. To protect privacy and prevent breaches of confidentiality, all study procedures starting with recruitment and extending through to findings dissemination will adhere to all site and IRB policies and applicable regulations. All study staff realize the importance of keeping private personal information secure and are trained biennially at the sites to minimize this risk. All study personnel will demonstrate knowledge of these regulations by completing courses of instruction through the CITI program and local HIPAA compliance offices.

Any consented photography or video will protect participants’ identity (e.g., by obscuring the subject’s face and any identifying marks like tattoos). The videos and photos may include the participant’s entire body, but they will be anonymized during data processing; the participant’s face will be blurred, any identifying marks will be covered or blurred, and then the original file will be deleted. If a participant’s voice is accidently recorded, that section of video would be altered prior to any use outside of the study team. The video camera and the recording media (e.g., SD cards, optical disks) will be stored in a locked office at the site. Photos and videos that do not contain identifiable information may also be stored on password-protected computers for future use in scientific presentations and publications.

Participant data will be collected at each site will be entered into independent and secure database. Only coded information will be entered, if necessary. In general, electronic data will be coded with links stored separately, stored on password protected drives, servers will be backed up regularly, firewalls in place, and access permitted only by study team members listed on the approved IRB protocol. All physical records pertaining to a participant’s involvement in this research study will be stored in a locked room within a locked filing cabinet. A participant number will indicate the participant’s identity on these records. A master sheet linking participant names and their participant numbers will be kept secure on a password protected computer by the principal investigator. Informed consent documents will be stored separately along with other identifiable information (such as medical records necessary to have in paper form, which will be limited). Consent documents and HIPAA authorizations will be kept for a minimum of 6 years following study closure. Paper files will be stored in a locked facility to which only study investigators and study team members will have access. Because this is a multi-site study, extra care will be taken not to share protected health information or other identifiable information about participants across sites. Upon completion of the research project, in accordance with local policy, we will ensure that study data be removed from all servers, desktops, removable storage devices, etc. Hard copy data with identifiable and/or sensitive information will be shredded. Electronic data containing identifiable information will be wiped using approved software.

Access to the data for processing and participant tracking will be limited to members of the research team. Coded data files may be sent off-site via DoD Safe or encrypted email to our biostatistician, off-site collaborators, between study investigators, and to the public according to established local data transmission guidelines and in accordance with procedures monitored by the IRB and the local Research and Development and Privacy Officer. Although every effort will be made to conduct study procedures in private, some subject interactions may, in a manner consistent with standard clinical assessment, take place in a common area, like a clinical treatment space where other patients may be receiving care. The study team will use standard practices to preserve the privacy of the participant as much as possible, such as refraining from discussing sensitive topics, discussing being in a research study, referring to private health information or other personally identifiable information.

All participant information will be handled in a confidential manner consistent with HIPAA policies. However, in unusual cases, the research records may be inspected by appropriate government agencies or be released in response to an order from a court of law. The investigators acknowledge that representatives of HJF and USAMRMC will also be eligible to review study records. Identifiable protected health information such as name, patient identifying number (required to identify an individual’s medical records), and contact information will be secured in a password protected electronic database at each site. This database will also contain the participant’s de- identified alpha-numeric code. As part of the data transfer plan, data will be shared between sites using a secure and approved platform such as encrypted email or the DoD SAFE web application. This encrypted Military file transfer system allows only designated recipients to download files. If SAFE becomes unusable at any point in time during the study, the investigative team will use the DoD’s or VA’s designated replacement program. Demographic and injury related identifiable information will be maintained and stored in a secure place according to institutional regulatory guidelines.

There are no plans to share data with providers outside the study team. The Principal Investigator and other members of the immediate study team (Research Coordinator, Research Assistant) will have access to study records and data. Beyond this, study results will be shared with the scientific community. The results of this study may be used for teaching, research, publications, or presentations at scientific meetings. When the results of this research are published or discussed in conferences, no information will be included that would reveal a subject’s identity. If a subject’s individual results are discussed, their identity will be protected by using a unique subject identifier rather than their name or other identifying information. Examples of identifying information may include medical record number, Social Security number, or address. The results of this study may be published, but subject records will not be revealed unless required by law.

# References

1. Armed Forces Health Surveillance Center, *Ambulatory Visits Among Members of the Active Component,*

*U.S. Armed Forces, 2010*. 2011: Medical Surveillance Monthly Report (MSMR). p. 16-21.

1. Owens, B.D., et al., *Extremity trauma research in the United States Army.* J Am Acad Orthop Surg, 2006.

**14**(10 Spec No.): p. S37-40.

1. Masini, B.D., et al., *Resource Utilization and Disability Outcome Assessment of Combat Casualties From Operation Iraqi Freedom and Operation Enduring Freedom.* J Orthop Trauma, 2009. **23**(4): p. 261-266.
2. Owens, B.D., et al., *Characterization of extremity wounds in operation Iraqi freedom and operation enduring freedom.* J Orthop Trauma, 2007. **21**(4): p. 254-257.
3. Fergason, J., J.J. Keeling, and E.M. Bluman, *Recent Advances in Lower Extremity Amputations and Prosthetics for the Combat Injured Patient.* Foot Ankle Clin North Am, 2010. **15**(1): p. 151-174.
4. Doukas, W.C., et al., *The Military Extremity Trauma Amputation/Limb Salvage (METALS) study: outcomes of amputation versus limb salvage following major lower-extremity trauma.* J Bone Joint Surg Am, 2013. **95**(2): p. 138-45.
5. *AOPA Fact Sheet [*Accessed 8 Aug 2018]; Available from: [http://www.aopanet.org/media/fact-sheet.](http://www.aopanet.org/media/fact-sheet)
6. DaVanzo, J., et al. *Projecting the adequacy of workforce supply to meet patient demand*. 2015 [Accessed

1 Aug 2018]; Available from: <http://www.ncope.org/view/?file=Dobson_DaVanzo_Final_Report_->

_NCOPE_Workforce_Demand_OP_Professionals_5.27.15.

1. Sumiya, T., Y. Suzuki, and T. Kasahara, *Stiffness control in posterior-type plastic ankle-foot orthoses: Effect of ankle trimline .2. Orthosis characteristics and orthosis/patient matching.* Prosthet Orthot Int, 1996. **20**(2): p. 132-137.
2. Bregman, D.J.J., et al., *The effect of ankle foot orthosis stiffness on the energy cost of walking: A simulation study.* Clinical Biomechanics, 2011. **26**(9): p. 955-961.
3. Collins, S.H., M.B. Wiggin, and G.S. Sawicki, *Reducing the energy cost of human walking using an unpowered exoskeleton.* Nature, 2015. **522**(7555): p. 212-+.
4. Ploeger, H.E., et al., *Stiffness modification of two ankle-foot orthosis types to optimize gait in individuals with non-spastic calf muscle weakness - a proof-of-concept study.* J Foot Ankle Res, 2019. **12**: p. 41.
5. Kerkum, Y.L., et al., *The Effects of Varying Ankle Foot Orthosis Stiffness on Gait in Children with Spastic Cerebral Palsy Who Walk with Excessive Knee Flexion.* PLoS One, 2015. **10**(11): p. e0142878.
6. Totah, D., et al., *The impact of ankle-foot orthosis stiffness on gait: A systematic literature review.* Gait Posture, 2019. **69**: p. 101-111.
7. Schaffalitzky, E., et al., *Understanding the benefits of prosthetic prescription: exploring the experiences of practitioners and lower limb prosthetic users.* Disabil Rehabil, 2011. **33**(15-16): p. 1314-23.
8. Van der Linde, H., et al., *From satisfaction to expectation: the patient's perspective in lower limb prosthetic care.* Disabil Rehabil, 2007. **29**(13): p. 1049-55.
9. Carr, A.J. and J.L. Donovan, *Why doctors and patients disagree.* Br J Rheumatol, 1998. **37**(1): p. 1-4.
10. Witte, K.A., A.M. Fatschel, and S.H. Collins, *Design of a lightweight, tethered, torque-controlled knee exoskeleton.* IEEE Int Conf Rehabil Robot, 2017. **2017**: p. 1646-1653.
11. Witte, K.A. and S.H. Collins, *Design of lower-limb exoskeletons and emulator systems*, in *Wearable Robotics: Systems and Applications*, P.W. Fergauson and J. Rosen, Editors. 2019, Elsevier. p. 251-274.
12. Witte, K.A., et al., *Improving the energy economy of human running with powered and unpowered ankle exoskeleton assistance.* Science Robotics, 2020. **5**: p. eaay9108.
13. Mahon, E., et al., *Barriers to Clinical Trial Recruitment and Possible Solutions: A Stakeholder Survey*, in

*Applied Clinical Trials*. 2015.

1. *Centers for Medicare and Medicaid Services. U.S. Department of Health and Human Services. Healthcare Common Procedure Coding System.* 2007, U.S. Department of Commerce, National Technocal Information Service: Springfield (VA).
2. Russell Esposito, E., Rodriguez, K.M., Rabago, C.A., and Wilken, J.M., *Does unilateral transtibial amputation lead to greater metabolic demand during walking.* JRRD, 2014. **51**(8): p. 1287-96.
3. Borg, G.A., *Perceived exertion.* Exerc Sport Sci Rev, 1974. **2**: p. 131-53.
4. Borg G. *Borg’s Perceived Exertion and Pain Scales*. Human Kinetics, 1998; p. 104.
5. Amtmann, D., et al., *Development of a PROMIS item bank to measure pain interference.* Pain, 2010.

**150**(1): p. 173-82.

1. Lajoie, Y. and S.P. Gallagher, *Predicting falls within the elderly community: comparison of postural sway, reaction time, the Berg balance scale and the Activities-specific Balance Confidence (ABC) scale for comparing fallers and non-fallers.* Arch Gerontol Geriatr, 2004. **38**(1): p. 11-26.
2. Sions, J.M., et al., *Balance-confidence is associated with community participation, perceived physical mobility, and performance-based function among individuals with a unilateral amputation.* Physiother Theory Pract, 2018: p. 1-8.
3. Mandel, A., et al., *Balance confidence and activity of community-dwelling patients with transtibial amputation.* J Rehabil Res Dev, 2016. **53**(5): p. 551-560.
4. Demers, L., R. Weiss-Lambrou, and B. Ska, *Development of the Quebec User Evaluation of Satisfaction with assistive Technology (QUEST).* Assist Technol, 1996. **8**(1): p. 3-13.
5. Lansade, C., et al., *Mobility and satisfaction with a microprocessor-controlled knee in moderately active amputees: A multi-centric randomized crossover trial.* Ann Phys Rehabil Med, 2018. **61**(5): p. 278-285.
6. Bettoni, E., et al., *A systematic review of questionnaires to assess patient satisfaction with limb orthoses.* Prosthet Orthot Int, 2016. **40**(2): p. 158-69.
7. Heinemann, A.W., R.K. Bode, and C. O'Reilly, *Development and measurement properties of the Orthotics and Prosthetics Users' Survey (OPUS): a comprehensive set of clinical outcome instruments.* Prosthet Orthot Int, 2003. **27**(3): p. 191-206.
8. Bohannon, R.W., Y.C. Wang, and R.C. Gershon, *Two-minute walk test performance by adults 18 to 85 years: normative values, reliability, and responsiveness.* Arch Phys Med Rehabil, 2015. **96**(3): p. 472-7.
9. Borg, G.A., *Psychophysical bases of perceived exertion.* Med Sci Sports Exerc, 1982. **14**(5): p. 377-81.
10. Pin, T.W., *Psychometric properties of 2-minute walk test: a systematic review.* Arch Phys Med Rehabil, 2014. **95**(9): p. 1759-75.
11. Ylva, N. and F. Anette, *Psychometric properties of the Activities-Specific Balance Confidence Scale in persons 0-14 days and 3 months post stroke.* Disabil Rehabil, 2012. **34**(14): p. 1186-91.
12. Forsberg, A. and Y. Nilsagard, *Validity and Reliability of the Swedish Version of the Activities-specific Balance Confidence Scale in People with Chronic Stroke.* Physiother Can, 2013. **65**(2): p. 141-7.
13. Tyson, S.F. and R.M. Kent, *Effects of an ankle-foot orthosis on balance and walking after stroke: a systematic review and pooled meta-analysis.* Arch Phys Med Rehabil, 2013. **94**(7): p. 1377-85.
14. Dite, W. and V.A. Temple, *A clinical test of stepping and change of direction to identify multiple falling older adults.* Arch Phys Med Rehabil, 2002. **83**(11): p. 1566-71.
15. Wilken, J.M., et al., *Physical performance assessment in military service members.* J Am Acad Orthop Surg, 2012. **20 Suppl 1**: p. S42-7.
16. Sawers, A. and B. Hafner, *Validation of the Narrowing Beam Walking Test in Lower Limb Prosthesis Users.* Arch Phys Med Rehabil, 2018. **99**(8): p. 1491-1498 e1.
17. Sawers, A. and B.J. Hafner, *Narrowing beam-walking is a clinically feasible approach for assessing balance ability in lower-limb prosthesis users.* J Rehabil Med, 2018. **50**(5): p. 457-464.
18. Bedigrew, K.M., et al., *Can an Integrated Orthotic and Rehabilitation Program Decrease Pain and Improve Function After Lower Extremity Trauma?* Clin Orthop Relat Res, 2014.
